# Fabrication of Sustained Release Curcumin-Loaded Solid Lipid Nanoparticles (Cur-SLNs) as a Potential Drug Delivery System for the Treatment of Lung Cancer: Optimization of Formulation and In Vitro Biological Evaluation

**DOI:** 10.3390/polym15030542

**Published:** 2023-01-20

**Authors:** Mohammad Akhlaquer Rahman, Abuzer Ali, Mohamed Rahamathulla, Shahana Salam, Umme Hani, Shadma Wahab, Musarrat Husain Warsi, Mohammad Yusuf, Amena Ali, Vineet Mittal, Ranjit Kumar Harwansh

**Affiliations:** 1 Department of Pharmaceutics and Industrial Pharmacy, College of Pharmacy, Taif University, P.O. Box 11099, Taif 21944, Saudi Arabia; 2 Department of Pharmacognosy, College of Pharmacy, Taif University, P.O. Box 11099, Taif 21944, Saudi Arabia; 3 Department of Pharmaceutics, College of Pharmacy, King Khalid University, P.O. Box 62236, Abha 62223, Saudi Arabia; 4 Department of Pharmaceutical Chemistry, College of Pharmacy, Prince Sattam Bin Abdulaziz University, P.O. Box 173, Al-Kharj 11942, Saudi Arabia; 5 Department of Pharmacognosy, College of Pharmacy, King Khalid University, P.O. Box 62236, Abha 62529, Saudi Arabia; 6 Department of Clinical Pharmacy, College of Pharmacy, Taif University, P.O. Box 11099, Taif 21944, Saudi Arabia; 7 Department of Pharmaceutical Chemistry, College of Pharmacy, Taif University, P.O. Box 11099, Taif 21944, Saudi Arabia; 8 Department of Pharmaceutical Sciences, Maharshi Dayanad University, Rohtak 124001, India; 9 Institute of Pharmaceutical Research, GLA University, Mathura 281406, India

**Keywords:** curcumin, solubility, lung cancer, solid lipid nanoparticle, erythrocyte toxicity, cytotoxicity, cellular uptake

## Abstract

The goal of current research was to develop a new form of effective drug, curcumin-loaded solid lipid nanoparticles (Cur-SLNs) and test its efficacy in the treatment of lung cancer. Different batches of SLNs were prepared by the emulsification–ultrasonication method. For the optimization of formulation, each batch was evaluated for particle size, polydispersity index (PI), zeta potential (ZP), entrapment efficiency (EE) and drug loading (DL). The formulation components and process parameters largely affected the quality of SLNs. The SLNs obtained with particle size, 114.9 ± 1.36 nm; PI, 0.112 ± 0.005; ZP, −32.3 ± 0.30 mV; EE, 69.74 ± 2.03%, and DL, 0.81 ± 0.04% was designated as an optimized formulation. The formulation was freeze-dried to remove excess water to improve the physical stability. Freeze-dried Cur-SLNs showed 99.32% of drug release and demonstrated a burst effect trailed by sustained release up to 120 h periods. The erythrocyte toxicity study of Cur-SLNs and its components demonstrated moderate hemolytic potential towards red blood cells (RBCs). The cytotoxic potential of the formulation and plain curcumin was estimated using 3-(4,5-dimethylthiazol-2-yl)-2,5-diphenyltetrazolium bromide (MTT) assay against A549 cell line. After 48 h of incubation, Cur-SLNs demonstrated more cytotoxicity (IC_50_ = 26.12 ± 1.24 µM) than plain curcumin (IC_50_ = 35.12 ± 2.33 µM). Moreover, the cellular uptake of curcumin was found to be significantly higher from Cur-SLNs (682.08 ± 6.33 ng/µg) compared to plain curcumin (162.4 ± 4.2 ng/µg). Additionally, the optimized formulation was found to be stable over the period of 90 days of storage. Hence, curcumin-loaded SLNs can be prepared using the proposed cost effective method, and can be utilized as an effective drug delivery system for the treatment of lung cancer, provided in vivo studies warrant a similar outcome.

## 1. Introduction

Cancer is one of the most life-threatening diseases that develop over a long stretch of time, causing millions of mortalities across the globe [1]. Recent cancer statistics have suggested a significant rise in the number of cancer patients, indicating breast cancer (11.7%) followed by lung (11.4%), colorectal (10.0%), prostate (7.3%), and stomach (5.6%) cancers [2]. Lung cancer is the 2nd most common cancer in men and women after prostate and breast cancer, respectively [3]. Moreover, lung cancer is the leading cause of death for men and women and is still the biggest reason of mortality amongst men all around the globe. There were more than 2.2 million new cases of lung cancer in 2020. The majority of lung cancer patients are detected when the disease has progressed to the point where surgical resection is no longer an option. Despite the fact that these patients are treated with standard chemotherapeutic medicines, their prognosis is poor, with a 5-year survival rate of only 15% [4]. Due to the development of cellular resistance, traditional chemotherapy has been ineffective in cancer treatment [5,6]. Furthermore, traditional chemotherapy and advanced molecular drug therapy are both expensive and inconvenient for patients [7]. Recent advancements in conventional medicine research have cleared the road for the development of innovative chemotherapy adjuncts. For decades, herbal derived-medicines have been used to treat a variety of ailments including cancer. Curcumin (Cur), a polyphenolic curcuminoid compound derived from the rhizomes of the plant *Curcuma longa*, popularly known as turmeric. Curcumin possesses numerous pharmacological properties including anti-inflammatory, antioxidant [8], antimicrobial [9], anticancer [10,11,12,13], and many more activities reported in the literature [14]. The capability of curcumin to affect a variety of targets including transcription factors, growth regulators, angiogenesis regulators, apoptotic genes, adhesion molecules, and cellular signaling molecules, is believed to be the basis of its anticarcinogenic properties [15]. Despite the fact that curcumin retain a wide range of pharmacological activities, its clinical application is limited due to its low water solubility (approximately 11 ng/mL), rapid metabolism, and degradation at different physiological pH conditions [16]. Therefore, there is a need to develop a robust formulation that can overwhelm the biopharmaceutical weaknesses associated with this molecule [17]. The application of nanotechnology in formulation can be employed to discourse the issues mentioned above [18,19].

Different types of nanoparticles derived from polymers, lipids, or metals have proven to be useful in diverse medical applications, from diagnosis to cancer therapy [20,21,22]. In an investigation, curcumin nanoparticles demonstrated a considerable antiproliferative effect against lungs, liver, and skin cancer cells [23], higher absorption in prostate cancer and human embryonic kidney cell line compared to plain curcumin. Furthermore, curcumin nanoformulation demonstrated significant inhibition of growth on colorectal cancer cell line mainly due to increased cellular uptake and protein binding [24]. Among different types of nanoparticles, solid lipid nanoparticles (SLNs) are the latest offshoot in the field of nanotechnology. The SLNs accompanied by biodegradable formulation components, can be safely loaded with therapeutic compounds for effective drug delivery with sustained release potential [21]. SLNs offer excellent biocompatibility, physical stability, high drug loading, physiological protection in gastrointestinal milieu, and enhanced cellular uptake [17,25]. Furthermore, feasibility for large scale production is the key advantage of SLNs [26,27]. However, it is important to note that the formulation components including lipid concentration (LC), surfactant concentration (SC) and drug concentration (DC) show remarkable influence on physicochemical properties of nanoparticles and its in vivo performances as well. Besides that, the manufacturing process parameters such as homogenization time (HT) and sonication time (ST) also affect the quality of final product in different ways. In this study, both formulation and process parameters were taken into consideration for a better-quality product. Optimization of formulation was achieved by evaluating each batch for their particle size, polydispersity index (PI), zeta potential (ZP), entrapment efficiency (EE) and drug loading (DL). The freeze-dried nanoformulation was subjected for in vitro drug release and erythrocyte toxicity studies. Further, therapeutic efficacy of the formulation was evaluated by cytotoxicity and cellular uptake studies using lung cancer cell line (A549).

## 2. Experimental Section

### 2.1. Materials

Curcumin was generously supplied by Loba chemicals, Banglore, India. Tween 80 was procured from Merck India Ltd. Glyceryl monostearate (GSM) was kindly delivered by Gattefosse, France. Trehalose was delivered as a gift sample from Sigma Aldrich, Inc., St. Louis, MI, USA. Dialysis membrane (MWCO: 1200 g/mole) was purchased from HiMedia, Mumbai, India. Human lung cancer cell line (A549) was procured from the American Culture Collection. The rest of the chemicals used were of analytical reagent grade.

### 2.2. Methods

#### 2.2.1. Methods for Lipid and Surfactant Selection

The method for the solubility study was adopted according to the previous report [28] with slight modification. In brief, 5 mg curcumin was added to a 10 mL capacity glass vial previously filled with a water/lipid mixture (1:1, *w*/*w*). Then, the glass vials were fixed in a water bath maintaining the temperature at 70 °C with continuous shaking for 1 h. The whole content was allowed to cool as a result the aqueous layer separated out. It was further centrifuged at 15,000 rpm for 15 min to settle any drug particles suspended. The aqueous layer was then analyzed for the amount of curcumin present using spectrophotometer at 430 nm wavelength using double distilled water as blank. The amount of curcumin present in aqueous layer was subtracted from the total amount of drug added gives solubility of curcumin expressed in percentage. Surfactant is one of the important components of SLNs that play an important role in the quality of nanoparticles. For the selection of appropriate surfactant, dummy formulation was prepared. In a 10 mL capacity volumetric flask, an accurately weighed solid lipid (5%) was added to a different surfactant (2%) in distilled water and slightly heated using a water bath maintained at 70 °C temperature. The mixture was vortexed followed by sonication for 5 min. Then, the mixture was immediately placed in an ice bath that leads to the formation of lipid dispersion. The lipid dispersion formed was examined for particle size, ZP, and PI. After storage for a period of 24 h, the lipid dispersion was examined for any precipitation formed.

#### 2.2.2. Optimization of Manufacturing Process and Formulation Components

**Figure 1:**
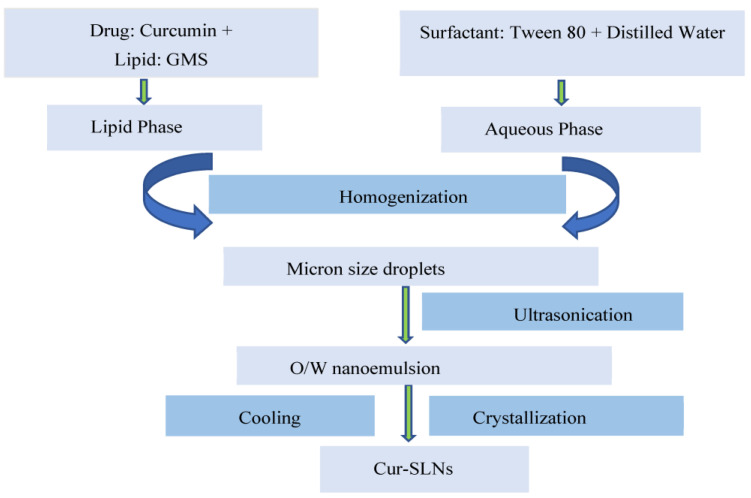
Schematic representation for the preparation of curcumin loaded solid lipid nanoparticles (Cur-SLNs).

The emulsification–ultrasonication method was used to prepare SLNs [29]. In brief, solid lipid having highest solubility for curcumin was placed in a volumetric flask and melted using hot water bath maintained at a temperature of 80 °C. Then, the surfactant was separately dissolved in distilled water in another volumetric flask. Both the lipid and aqueous phases obtained above were maintained at the same temperature and then mixed with each other. The mixture was blended properly with the help of a high-speed homogenizer maintained at a speed of 5000 rpm. The mixing of the lipid and aqueous phase leads to the formation of lipid dispersion with micron-sized droplets. Further, the lipid dispersion was ultrasonicated with the help of a probe sonicator to break the micron-sized droplets into nano-sized droplets (*o*/*w* nanoemulsion). The *o*/*w* nanoemulsion obtained was rapidly allowed to cool in an ice water bath. The process of rapid cooling initiated crystallization of the lipid and leads to the formation of SLNs. Figure 1 represents the steps for the preparation of SLNs. Optimized formulation was achieved by changing the critical process variables, as mentioned in schemes 1–5.

Scheme 1, Homogenization time (HT, min): 1, 2, 5, 10; Sonication time (ST, min): 10; Surfactant concentration (SC, % *w*/*v*): 2.5; Lipid concentration (LC, % *w*/*v*): 5; Drug concentration (DC, % *w*/*v*): 1;

Scheme 2, Homogenization time (HT, min): 5; Sonication time (ST, min): 1, 2, 5, 10; Surfactant concentration (SC, % *w*/*v*): 2.5; Lipid concentration (LC, % *w*/*v*): 5; Drug concentration (DC, % *w*/*v*): 1;

Scheme 3, Homogenization time (HT, min): 5; Sonication time (ST, min): 10; Surfactant concentration (SC, % *w*/*v*): 1, 2, 2.5, 3%, Lipid concentration (LC, % *w*/*v*): 5; Drug concentration (DC, % *w*/*v*): 1;

Scheme 4, Homogenization time (HT, min): 5; Sonication time (ST, min): 10; Surfactant concentration (SC, % *w*/*v*): 2.5; Lipid concentration (LC, % *w*/*v*): 1, 2.5, 5, 10; Drug concentration (DC, % *w*/*v*): 1;

Scheme 5, Homogenization time (HT, min): 5; Sonication time (ST, min): 10; Surfactant concentration (SC, %, *w*/*v*): 2.5; Lipid concentration (LC, % *w*/*v*): 5; Drug concentration (DC, % *w*/*v*): 0, 0.5, 1, 1.5, 2, 2.5

In order to achieve a solid-state product from the lipid nanoformulation, freeze-drying was performed using a freeze-dryer (Biocryos, Gyeonggi-do, Korea). Freeze-drying facilitated the removal of excess water from the optimized lipid nanoformulation. The freeze-dried product exhibits improved and extended stability with least tendency for particle aggregation. Trehalose was added as a cryoprotectant into the lipid nanoformulation before the start of the drying process. Trehalose was added at the concentration level between 5 and 10% *w*/*v*. At this concentration level, trehalose was supposed to demonstrate their efficiency towards preserving the physicochemical properties of nanoparticles formed. Initially, slow freezing was carried out (−40 °C, 2 h) followed by freezing with an increasing rate of 5 °C/h from −40 °C to 25 °C till the end of 24 h. The dried SLNs were used for drug release, and biological performances including erythrocyte toxicity, cytotoxicity and cellular uptake.

#### 2.2.3. Particle Size and Zeta Potential Measurement

Photon correlation microscopy was used to measure the average particle size and PI of nanoparticles. The particle size was measured using Malvern Zetasizer 4 (Malvern Instruments, UK). The instrument was equipped with a He-Ne laser at a fixed angle of 90°. Before starting the analytical procedure, the sample was suitably diluted (1:100) with double distilled water and the experiment was performed in triplicate. The number of runs and the applied voltage was determined automatically by the software. The surface charge/zeta potential was calculated from electrophoretic mobility. The zeta potential gives information about the possibility of nanoparticles aggregation [30].

#### 2.2.4. Transmission Electron Microscopy (TEM)

The surface morphology of Cur-SLNs were examined with the help of transmission electron microscopy (TEM) (Techni TEM 200 kV)). A drop of freshly prepared Cur-SLNs suspension was placed onto a carbon-layered copper grid and air-dried. After negative staining with a drop of phosphotungstic acid (1%), images of the SLNs were viewed.

#### 2.2.5. Entrapment Efficiency and Drug Loading

The drug extraction method was used to determine the entrapment efficiency of SLNs. In brief, 10 mg of nanoformulation was first dissolved in 1 mL of 1-octanol and then mixed with 5 mL phosphate buffer (pH 6.8) in a beaker with vigorous shaking for at least 5 min. The drug was allowed to partition in both aqueous and lipid phase for a period of 2 h. After 2 h of standing, phase separation takes place, i.e., the aqueous phase separated from lipid phase. The aqueous layer was removed, and absorbance was taken using spectrophotometer at a wavelength of 430 nm. The amount of drug present in the aqueous layer was determined with the help of calibration standard. The total amount of the drug added in the formulation and the actual amount of the drug in the nanoformulation was used to calculate entrapment efficiency (EE%) and drug loading (DL%) using equation:

(1) $$\mathrm{Entrapment}\;\mathrm{efficiency}\;\left(\%\right)=\frac{\text{Amount of curcumin in nanoformulation}}{\text{Total amount of curcumin added}}\times 100$$

(2) $$\mathrm{Drug}\;\mathrm{loading}\;\left(\%\right)=\frac{\text{Amount of curcumin in nanoformulation}}{\text{Total amount of curcumin}+\text{Total amount of lipid}}\times 100$$

#### 2.2.6. In Vitro Release Study

The dialysis bag method was used for in vitro release study. The experiment was performed to quantify the release of curcumin from SLNs and unformulated curcumin/plain curcumin. Dialysis bag (MWCO: 1200 g/mole) and USP dissolution apparatus (Type II) was used for the study. Phosphate buffer saline (PBS) adjusted to pH 7.4 was used as dissolution media. Temperature of the dissolution media and paddle speed were maintained at 37 ± 0.5 °C and 100 rpm, respectively. Before starting the process, the dialysis bag was pre-treated/soaked in double distilled water and left overnight. The pre-treated dialysis bag was individually filled with 0.5 g SLNs and plain curcumin. Then, the bag was tied at both ends and placed in 900 mL of dissolution media kept in the glass jar of 1000 mL capacity. A 0.5 mL sample was taken out at a fixed time interval of 0, 1, 2, 4, 8, 12, 24, 48, 72, 96 and 120 h. The sink condition was maintained by replacing the sample with the same dissolution media. The samples were filtered with 0.45 µm filter paper and the amount of drug released was determined spectrophotometrically at a wavelength of 430 nm.

#### 2.2.7. Erythrocyte Toxicity Study

The SLNs and its formulation ingredients may cause hemolysis of red blood cells (RBCs). Therefore, the hemolytic potential of the formulation and its individual components had to be determined. Fresh human blood was required for the study. A written consent was taken from the donor prior to the collection of blood. A 10 mL sample of fresh blood was utilized to obtain the erythrocyte pellets. The erythrocyte pellets were diluted with 0.9% NaCl solution to obtain dispersion of RBCs pellets (3%, *w*/*v*). The dispersion of RBCs pellets was stored in a refrigerator at a temperature of −21 °C for a period of 24 h. The required quantity of optimized formulation (Cur-SLNs), plain curcumin solution, and individual formulation components (glyceryl monostearate, tween 80, and trehalose) were added to 1 mL of dispersion. The volume was adjusted to 5 mL with 0.9% NaCl solution to achieve a final concentration of 50 µg/mL. All the samples were incubated at 37 °C for 2 h. After 2 h of storage, samples were centrifuged at 2000 rpm for a fixed time period of 10 min. After centrifugation, a clear transparent supernatant was removed, and absorbance was taken at a wavelength of 430 nm using a UV spectrophotometer. Triton X-100 was used as a positive control and 0.9% NaCl as a negative control. The % hemolysis was calculated using the formula [31,32].

(3) $$\mathrm{Hemolysis}\;\mathrm{of}\;\mathrm{RBCs}\;\left(\%\right)=100\times \frac{\left(\text{UV absorbance of test sample}\right)-\left(\text{UV absorbance of negative control}\right)}{\left(\text{UV absorbance of positive control}\right)-\left(\text{UV absorbance of negative control}\right)}$$

#### 2.2.8. Cytotoxicity Potential of Curcumin

A 3-(4,5-dimethylthiazol-2-yl)-2,5-diphenyltetrazolium bromide (MTT) assay was used to assess the viability of lung cancer cells. To execute the whole process, lung cancer cells (A549, ATCC^®^ CCL-185™) was cultured in RPMI 1640 medium composed of 100 U/mL penicillin-streptomycin and 10% fetal bovine serum. The culture was grown at 5 × 10^4^ cells/well into 96-well plates and was allowed to attach overnight at a temperature of 37 °C in an atmosphere fully saturated with water and supplemented with 5% carbon dioxide (CO_2_). Fresh culture media was used for the preparation of plain curcumin suspension and Cur-SLNs suspension. The samples were dissolved in dimethyl sulfoxide maintaining its concentration < 1%, *v*/*v*. These solutions were applied in a series of doses on A549 cells plated into 96-well plates with a density of approximately 5000 cells/well and incubated for 48 h. The untreated cells were used as negative control, and 0.5% DMSO were run for each 96-well plate as an untreated control. Then, into the above-prepared cell culture medium, MTT reagent (20 µL) was added and kept into an incubator maintaining the required condition (temp. 37 °C; air supplement: 5% CO_2_, humidity: 95%, and time: 4 h). The solution was then transferred to 96-well plates and immediately read at a wavelength of 570 nm on a microplate reader (Bio-Rad, Hercules, CA, USA).

#### 2.2.9. Determination of Cellular Uptake of Curcumin

The in vitro cellular uptake of curcumin was carried out using A549 lung cancer cells. The total amount of curcumin present inside the cells was quantified to know the amount of drug uptake by A549 lung cancer cells. The cellular uptake was expressed as total drugs (ng) estimated in total protein (µg). A total of 5 × 10^4^ cells per well were taken for the study. The cells were inoculated overnight onto 24-well plates at a temperature of 37 °C. The cells were then exposed with pure curcumin and Cur-SLNs as a test sample corresponding to 20 µg/mL. The cells after exposure with the test sample were incubated for another 24 h at a temperature of 37 °C. After 24 h of incubation, the cells were washed with PBS and subjected to cell lysis. X-100 solution (1% *v*/*v*; 0.5 mL)/well) was used as lytic reagent to precipitate out proteins. The extraction of the drug from lysed cells was achieved by adding acetonitrile (0.5 mL/well) followed by centrifugation at 13,000 rpm for 10 min. Finally, the clear supernatant was removed and filtered through a 0.45 µm filter for analysis. The drug was quantified by high performance liquid chromatography (HPLC). The cellular uptake was calculated using the equation:

(4) $$\mathrm{Cellular}\;\mathrm{uptake}\;\mathrm{of}\;\mathrm{curcumin}=\frac{\text{Total amount of curcumin}\;(\mathrm{ng})\mathrm{estimated}}{\text{Total protein}\;(\mu g)}$$

#### 2.2.10. High-Performance Liquid Chromatography Analysis

High-performance liquid chromatography (HPLC, Shimadzu LC-10AT VP) was used for quantification of curcumin. A mixture of acetonitrile:water in 70:30 *v*/*v* was used as the mobile phase. The temperature of the column (C18 column, 25 cm × 4.6 mm, 5 mm particle size) was set to 40 °C and the flow rate of the mobile phase was 1.5 mL/min. The retention time for curcumin was 2.6 min. A total of 1 mL of each sample was used as volume of injection and analyzed at a wavelength of 430 nm with a UV detector (Shimadzu, Kyoto, Japan). A Class VP, version 5.032 software was used for data acquisition.

#### 2.2.11. Long-Term Stability Study

Long term stability study was performed at 25 °C /60% RH. The Cur-SLNs were filled in amber-colored glass bottles and kept in a stability chamber. At pre-determined time interval, the samples were withdrawn and evaluated for drug content and entrapment efficiency. The samples were also evaluated for physicochemical parameters including particle size, ZP and PI. For the determination of curcumin content in the samples withdrawn, the Cur-SLNs were disrupted in methanol:chloroform (1:1) and suitably diluted with the same solvent system. The analysis of the sample was performed spectrophotometrically at a wavelength of 430 nm. The content of curcumin was calculated using the following equation:

(5) $$\mathrm{Curcumin}\;\mathrm{content}\;\left(\%\right)=\frac{\text{Practical curcumin content}}{\text{Theoretical curcumin content}}\times 100$$

#### 2.2.12. Stability of Curcumin and Cur-SLNs in Phosphate Buffer

In order to ascertain the stability of plain curcumin and Cur-SLNs in PBS, pH 7.4, curcumin and Cur-SLNs solution were prepared in PBS at a fixed concentration and volume of 10 µg/mL and 5 mL, respectively. Both the samples were kept in a glass vial and incubated at 37 °C for 12 h. Plain curcumin was dissolved in PBS with the help of methanol (in concentration ≤ 5% *v*/*v*). At predetermined time points, 100 µL of solutions were taken and filtered with 0.45 µm filter paper and the amount of drug was determined spectrophotometrically at a wavelength 430 nm to quantify the stability of curcumin in PBS with time. The experiments were repeated three times for each time point (n = 3).

#### 2.2.13. Statistical Analysis

Data are expressed as the mean ± SD of at least three independent experiments. Raw data were evaluated using Graph Pad InStat demo version (GraphPad Software Inc., La Jolla, CA, USA). Statistical analysis was carried out using a one-way ANOVA along with Tukey–Kramer multiple comparison tests. A *p*-value of <0.05 was considered statistically significant.

## 3. Results

A solubility study was performed to select suitable lipid for the preparation of solid lipid nanoparticles. Glyceryl monostearate showed highest solubilization capacity for curcumin with percent drug partitioning values of 67.23% followed by palmitic acid (62.37%), stearic acid (57.34%), and cetostearyl alcohol (43.27%). Hence, glyceryl monostearate was selected as the lipid phase. Tween 80 was selected as a suitable surfactant because tween 80-based dummy SLNs demonstrated the smallest particle size (103.6 nm), an optimum PI (0.127), and ZP (−37.2 mV). The formulation was found to be stable towards any sign of precipitation after 24 h of storage.

Although the emulsification–ultrasonication method is fast and efficient, there are many factors that affect the quality of nanoparticles including homogenization time, sonication time, concentration of surfactant, lipid, and drug as well. Particle size, PI, ZP, EE, and DL of different batches of SLNs recorded with varying dependent factors are mentioned in Table 1.

**Figure 2:**
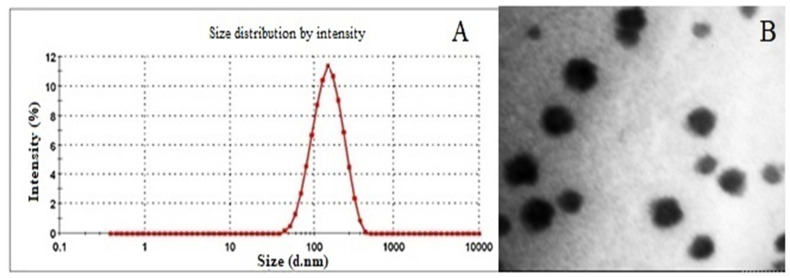
(**A**) Particle size (114.9 nm) of optimized formulation measured by Malvern Zetasizer 4 (Malvern Instruments). (**B**) TEM photograph of Cur-SLNs showing spherical shape and smooth surface.

Both the process parameters, homogenization and sonication time were varied from 1–10 min to optimize the formulation. HT and ST were optimized at 5 and 10 min, respectively. The formulation components, surfactant, lipid and drug concentration were also varied to optimize the percentage composition of each constituent. The optimized formulation with surfactant (2.5, % *w*/*v*), lipid (5, % *w*/*v*), and drug concentration (1, % *w*/*v*) demonstrated a particle size of 114.9 ± 1.36 nm (Figure 2A), PI: 0.112 ± 0.005, ZP: −32.3 ± 0.30 mV, EE: 69.74% ± 2.03 and DL: 0.81% ± 0.04. The TEM micrograph of Cur-SLNs is shown in Figure 2B. The shape of SLNs revealed spherical shape with a smooth surface. The SLNs were freeze-dried using trehalose. The particle size, PI, ZP, EE and DL of dried SLNs after reconstitution in double distilled water were 119.5 ± 2.17 nm, 0.124 ± 0.012, −34.6 ± 0.32 mV, 71.43 ± 1.53% and, 0.77 ± 0.03%, respectively (Table 2). The results demonstrated that the difference in all the parameters before and after drying were not statistically significant and hence used for further study.

**Figure 3:**
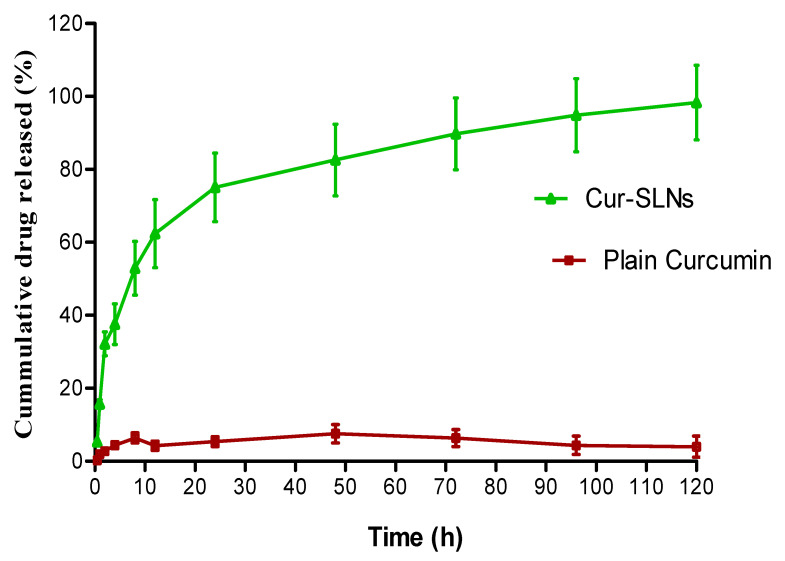
Cumulative % release of curcumin from Cur-SLNs in PBS (0.01 M, pH 7.4) and plain curcumin. Initial burst effect followed by sustained release effect up to 120 h.

**Figure 4:**
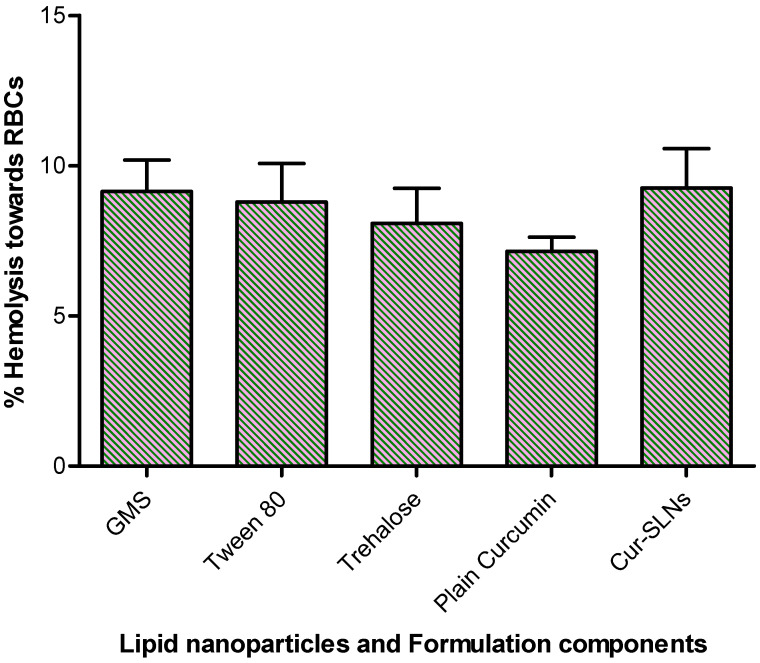
% hemolysis caused by Cur-SLNs, plain curcumin, formulation components (lipid: GMS, surfactant: tween 80, cryoprotectant: trehalose).

The in vitro release of curcumin from the formulation was performed using dialysis bag method. About 99% of curcumin was released from Cur-SLNs at the end of 120 h. On the other hand, only 10% of curcumin released from the pure drug demonstrated the enhanced solubility of curcumin (Figure 3). The hemolytic potential of the optimized Cur-SLNs and its formulation components are shown in Figure 4. The % hemolysis displayed by Cur-SLNs and plain curcumin solution was 9.26 ± 1.31% and 7.15 ± 0.47%, respectively, at a concentration of 50 µg/mL, while % hemolysis of individual components of the formulation was glyceryl monostearate (9.14 ± 1.05%), tween 80 (8.79 ± 1.28%), trehalose (8.08 ± 1.17%).

**Figure 5:**
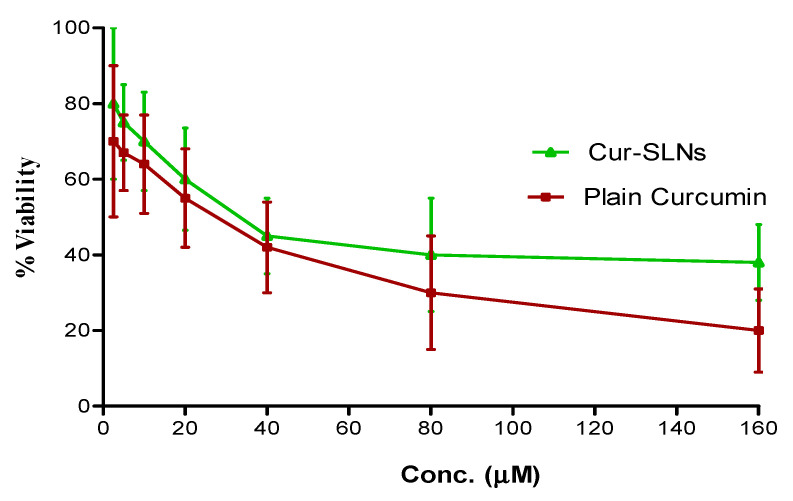
% Viability of curcumin and Cur-SLNs treated A549 cells for 24 h. IC_50_ value of Cur-SLNs and plain curcumin are 26.12 ± 1.24 µM and 26.12 ± 1.24 µM, respectively.

Figure 5 represents % viability with respect to curcumin concentration. When applied concentration increased during 24 h of incubation, cells exposed to various doses of curcumin showed enhanced inhibition in growth of A549 cells. The value of IC_50_ estimated for plain curcumin was 35.12 ± 2.33 µM. On the other hand, the IC_50_ value of Cur-SLNs was calculated as 26.12 ± 1.24 µM after 24 h of exposure. The effect of SLNs formulation on increasing curcumin uptake in A549 cells was investigated in a cellular uptake assay. The uptake of plain curcumin and Cur-SLNs was found to be 162.4 ± 4.2 ng/µg and 682.08 ± 6.33 ng/µg, respectively. The results demonstrated a 5.2-fold increase in cellular uptake of curcumin from Cur-SLNs than from plain curcumin.

**Figure 6:**
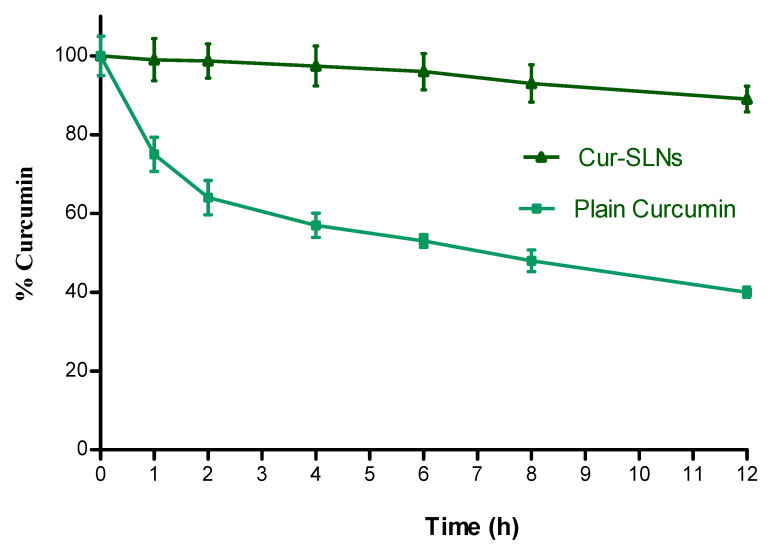
Stability of plain curcumin and Cur-SLNs in PBS (pH 7.4) at 37 °C. Data as mean ± SD, n = 3.

The particle size, PI, EE and drug content at 0, 30, 60, and 90 days of storage at 25 °C /60% RH demonstrated the results as follows; at 0 time; particle size: 119.5 ± 2.17 nm, PI: 0.124, ZP: (−) 34.6 ± 0.32 mV, EE: 71.43 ± 1.53%, at 30 days; particle size: 121.4 ± 2.48 nm, PI: 0.128, ZP: (−) 31.2 ± 0.27 mV, EE: 71.56 ± 2.17%, at 60 days; particle size: 124.7 ± 2.41 nm, PI: 0.139, ZP: (−) 29.6 ± 0.31 mV; EE: 73.14 ± 1.38%, and at 90 days: particle size: 126.6 ± 3.05 nm, PI: 0.147, ZP: (−) 28.7 ± 0.89 mV; EE: 77.23 ± 2.74%. The % curcumin content at 30, 60 and 90 days was found to be 99.34, 98.56, and 97.78%, respectively. Curcumin is a hydrophobic polyphenol compound that is unstable in aqueous solution. To confirm whether our formulation can increase curcumin’s solubility, we incubated plain curcumin and Cur-SLNs solution in PBS, pH 7.4 and estimated its concentration with time. As shown in Figure 6, after incubation for 12 h at 37 °C, plain curcumin in PBS degraded quickly and only 40% remained after the 12 h incubation. However, Cur-SLNs were stable in the same condition (89%).

## 4. Discussion

### 4.1. Lipid and Surfactant Selection

The purpose of the solubility study of curcumin in different lipids was to choose appropriate lipids in order to maximize drug loading and entrapment efficiency of SLNs. The drug molecules have an inherent property to partition into both aqueous and lipid phase. The partitioning behavior of curcumin between these phases was used for solubility measurement. Glyceryl monostearate and tween 80 was selected as suitable lipid and surfactant, respectively.

### 4.2. Homogenization and Sonication Time

The process of homogenization in formulation development is intended for emulsification of lipid phase in an aqueous phase. It does not play any role on the physicochemical properties of the final product. The ZP value is estimated to predict the physical stability of a nanoformulation that represents the strength of attraction between like-charged particles [33]. High ZP either with positive or negative values electrically stabilizes scattered nanoparticles. The stabilization of nanoparticles is principally due to electrical repulsion between similarly charged particles that lead to inhibition of agglomeration. ZP value was maximum (−32.3 mV) after 5 min of HT considered as stable nanoformulation. Both EE and DL of the nanoformulation was a bit higher after 5 min of HT, but there was no significant difference between the batches.

Sonication time is an important parameter in the formulation of SLNs as it helps in the reduction of micron-sized droplets into nano-sized droplets, leads to the formation of *o*/*w* nanoemulsion. Sonication time had a significant impact on PI too. The longer the ST, the smaller the nanoemulsion droplets and a lower PI, mainly due to more sonication energy delivered to split the SLN dispersions. In our study, sonication time demonstrated significant impact on both particle size and PI of nanoformulation that could be considered as a proof of facts. With increasing sonication time, both particle size and PI reduced dramatically. The difference in EE and DL between the batches was not statistically significant. In the batch with sonication time of 10 min, ZP was highest, indicating the maximum stability of nanoformulation.

### 4.3. Surfactant and Lipid Concentration

Surfactant concentration had a significant impact on the physicochemical properties of nanoparticles. The previous finding demonstrated that the particle size of the nanoformulation decreases considerably with an increase in the surfactant concentration [34], and similar results were achieved in our investigation. Surfactant concentration also affects the PI of the final product. Surfactant let down the surface tension of newly developed nanoparticles during the process of homogenization and prevent particle aggregation [35]. Similarly, low surfactant concentration causes instability and recrystallization of nanoparticles [36]. As observed, the PI decreased with an increase in surfactant concentration of up to 2.5%. The decrease in PI might be due to the presence of enough surfactant concentration on lipid droplets to keep the nanodroplets disaggregated and stable. Surprisingly, at 3% surfactant concentration, PI was higher than at 2.5%. Increase in surfactant concentration resulted in a considerable increase in EE and DL. This could be attributed to the presence of maximum surfactant concentration left over the surface of nanoformulation. The formulation with 2.5% surfactant had the highest ZP, indicating maximum stability of nanoformulation.

Lipid concentration has a remarkable effect on the size of nanoparticles. With an increase in lipid concentration, particle size also increased considerably. With increasing lipid concentration, a substantial rise in particle size was found. The dilute dispersion has a better sonication energy distribution than the concentrated dispersion, resulting in more efficient particle size reduction. Both particle size and PI were high when 10% lipid was used in the formulation. The EE significantly increased with increasing lipid concentration due to the availability of higher amount of lipid for drug encapsulation. On the other hand, drug loading reduced significantly with an increase in lipid concentration, which could be related to a lower drug-to-lipid ratio. The formulation with 5% lipid had the highest ZP, indicating stable nanoformulation.

### 4.4. Drug Concentration

Changing the drug concentration from 0.5 to 2.5% resulted in significant changes in particle size among different batches. At lower concentrations (up to 1.5%), there were no significant variations in particle size but at high concentrations (above 1.5%), particle size increased significantly. On the other hand, PI increased at higher drug concentration (1 to 2.5%). However, 0.5% drug concentration showed higher PI than 1 and 1.5%. At higher drug concentrations, the increase in particle size and PI was most likely attributable to the large quantity of drug present in the nanoformulation. A direct relationship between the added amount of the drug and EE was attained. An increase in drug concentration resulted in a considerable decrease in EE. With increasing drug concentration up to 2%, drug loading also increased. A further increase in drug concentration resulted in no additional increase in the capacity of drug loading. Results demonstrated that the drug loading has already reached its maximum value at 2% of drug concentration. ZP was highest in the formulation with 1% drug concentration, indicating stable nanoformulation.

### 4.5. Freeze-Drying of Nanoformulation

The physical and chemical stability of the formulation is essentially improved after lyophilization of nanoformulation and hence considered as an important step in formulation development. There are maximum chances of surfactant film disruption around nanoparticles during the drying process. Additionally, freeze-dried products may undergo particle aggregation after reconstitution or redispersion process [37]. Hence, the addition of cryoprotectant play an important role to prevent film disruption and aggregation of nanoparticles. Earlier, several reports already established the use of glucose, fructose, maltose, trehalose, sorbitol, and mannose as cryoprotectant to decrease nanoparticle aggregation due to the stress generated in the process of the freeze-drying process [38,39]. In our study, the mean particle size of cryoprotectant free freeze-dried SLNs drastically increased compared to that of un-lyophilized original dispersion. The results demonstrated that the nanoparticles obtained after freeze-drying with 5% trehalose preserve the physicochemical properties of nanoparticles (Table 2).

### 4.6. In Vitro Drug Release

The dialysis bag diffusion technique was used to investigate curcumin release from Cur-SLNs and unformulated/plain curcumin. The rate of curcumin release from the formulation and its appearance in the dissolution media was determined by the drug’s partition coefficients between the lipid phase and aqueous environment in the dialysis bag as well as by drug diffusion across the membrane. The dialysis bag retained the nanoparticles and enabled the diffusion of the drug immediately into dissolution media. To depict the drug release pattern, the % cumulative drug release vs. time is shown in Figure 3. The % cumulative release of curcumin from Cur-SLNs was much higher than from plain curcumin powder. The release of curcumin that were adsorbed on the surface of the nanoparticles triggered the Cur-SLNs to have a burst effect, with about 32.85% of the drug being released in the first 2 h followed by a sustained release phase for a further 120 h. The diffusion of drug molecules through the lipid matrix of SLNs into the dissolution media may be the cause of the sustained release effect. Similar results (initial burst effect followed by sustained release effect from a polymeric nanocarrier) have been reported in the literature [40], justify our findings.

### 4.7. Erythrocyte Toxicity

The erythrocyte toxicity caused by the formulation and its components are one of the important studies in the development of any drug delivery system. In fact, in vivo potential of formulation can be assessed by estimating the damage of erythrocyte in in vitro. Triton X-100 used as a positive control exhibited complete (100%) hemolysis, negative control (0.9% NaCl) showed no hemolysis (0%). The difference in % hemolytic exerted by plain curcumin, Cur-SLNs and its components were not statistically significant (*p* > 0.05). All the formulation components displayed biocompatibility with human blood and no additive effect on hemolysis of erythrocyte was observed. Furthermore, incorporation and assembly of all the ingredients together into SNLs does not show any additive effect on erythrocyte toxicity. Depending upon the results obtained, it may be suggested that the formulation can be suitably used for oral administration.

### 4.8. In Vitro Cytotoxicity and Cellular Uptake

Determination of cell viability is an important parameter for toxicity assay and is directly related with the toxic effects of active drug molecules [41]. Curcumin induced a decrease in cell viability in A549 cells. The decrease in cell viability was influenced by both drug concentration and cell density. Figure 5 represents % viability with respect to curcumin concentration. The growth of A549 cells was inhibited as a result of the short time treatment with Cur-SLNs. The Cur-SLNs exhibited more cytotoxicity against A549 cells than plain curcumin. The difference in cytotoxic effect was statistically significant (*p* < 0.01). The effect of SLNs formulation on increasing curcumin uptake in A549 cells was investigated in a cellular uptake assay. The uptake of curcumin from Cur-SLNs was 5.2-fold more than free curcumin. These findings could be linked to SLNs potential to enhance cellular absorption of a loaded drug owing to the presence of lipids [42].

### 4.9. Stability

The % increment in the particle size and EE was about 6 and 8% after 90 days of storage. The decrease in high surface to volume ratio may have contributed to partial aggregation, which led to an increase in particle size. EE was reduced due to the possibility that lipids could slowly change from their metastable to stable state during storage due to the small particle size [43]. The decrease in % curcumin content at the end of 90 days was only 2%. There was no significant change (*p* < 0.05) in particle size, EE, PI, ZP and % curcumin content. Hence, it could be concluded that SLNs were stable and robust in nature towards storage condition at 25 °C/60% RH. Moreover, it was noteworthy that the incorporation of curcumin in SLNs increased the stability of curcumin in PBS by protecting the encapsulated curcumin against hydrolysis and biotransformation.

## 5. Conclusions

In the present research envisaged, Cur-SLNs were successfully developed using the emulsification–ultrasonication method. The quality of the nanoformulation was considerably affected by almost all formulation components and manufacturing process parameters. The optimized nanoformulation demonstrated particle size, 114.9 ± 1.36 nm; PI, 0.112 ± 0.005; ZP, −32.3 ± 0.30 mV; EE, 69.74 ± 2.03%, and DL, 0.81 ± 0.04%. The Cur-SLNs were nanoscale in size, had a narrow size distribution, enough surface electrostatic charge to avoid aggregation with improved physical stability, high entrapment efficiency, and drug loading. TEM micrographs revealed a smooth surface and a spherical shape. Formulation showed a sustained release pattern for a period of 120 h. The incorporation of curcumin in lipid matrix of SLNs proficiently improved the cellular uptake of the drug by A549 cells. Both curcumin and Cur-SLNs were found to be cytotoxic to A549 cells in a dose-dependent manner, with the latter being more potent. Furthermore, Cur-SLNs were stable at 25 °C/60% RH for 3 months of the study period. The conclusive result of the study is that Cur-SLNs improved the therapeutic potential and expanded the current use of novel formulations of curcumin in the treatment of lung cancer, provided in vivo studies warrant similar outcomes. Hence, further in vivo studies are needed to validate these results.

## Figures and Tables

**Table 1:** Measured particle size, PI, ZP, EE, and DL of SLNs with varying process and formulation parameter.

Non-Variable Parameters	Variable Parameter	Particle Size (nm)	PI	ZP (−mV)	EE (%)	DL (%)
Scheme 1: ST (min): 10; SC (%, *w*/*v*): 2.5; LC (%, *w*/*v*): 5; DC (%, *w*/*v*): 1	HT: 1	124.6 ± 0.54	0.183 ± 0.012	24.5 ± 0.25	66.32 ± 1.53	0.76 ± 0.03
	HT: 2	125.7 ± 0.63	0.154 ± 0.014	23.4 ± 0.21	65.32 ± 1.46	0.78 ± 0.05
	HT: 5	114.9 ± 1.36	0.112 ± 0.005	32.3 ± 0.30	69.74 ± 2.03	0.81 ± 0.04
	HT:10	125.2 ± 0.71	0.203 ± 0.021	25.3 ± 0.13	64.68 ± 2.14	0.75 ± 0.02
Scheme 2: HT (min): 5; SC (%, *w*/*v*): 2.5; LC (%, *w*/*v*): 5; DC (%, *w*/*v*): 1	ST: 1	340.6 ± 3.12	0.434 ± 0.023	25.3 ± 0.34	66.16 ± 0.54	0.72 ± 0.01
	ST:2	285.4 ± 2.24	0.301 ± 0.046	27.2 ± 0.24	68.24 ± 1.34	0.74 ± 0.02
	ST:5	177.8 ± 1.44	0.162± 0.025	29.0 ± 1.18	68.13 ± 2.67	0.78 ± 0.04
	ST:10	114.9 ± 1.36	0.112 ± 0.005	32.3 ± 0.42	69.74 ± 2.03	0.81 ± 0.04
Scheme 3: HT (min): 5; ST (min): 10; LC (%, *w*/*v*): 5; DC (%, *w*/*v*): 1	SC: 1	224.3 ± 1.27	0.302 ± 0.025	28.3 ± 0.42	62.42 ± 1.86	0.71 ± 0.03
	SC: 2	142.7 ± 1.53	0.250 ± 0.007	29.2 ± 1.34	65.68 ± 1.43	0.76 ± 0.01
	SC: 2.5	114.9 ± 1.29	0.112 ± 0.005	32.3 ± 0.42	69.74 ± 2.03	0.81 ± 0.04
	SC: 3	102.4 ± 1.13	0.133 ± 0.027	25.5 ± 1.35	74.39 ± 2.63	0.83 ± 0.02
Scheme 4: HT (min): 5; ST (min): 10; SC (%, *w*/*v*): 2.5; DC (%, *w*/*v*): 1	LC: 1	58.6 ± 0.63	0.280± 0.025	22.4 ± 0.44	43.45 ± 1.62	0.97 ± 0.04
	LC: 2.5	81.5 ± 1.83	0.179 ± 0.019	26.2 ± 0.36	58.23 ± 2.32	0.88 ± 0.02
	LC: 5	114.9 ± 1.29	0.112 ± 0.005	32.3 ± 0.42	69.74 ± 2.03	0.81 ± 0.03
	LC: 10	231.4 ± 2.13	0.224 ± 0.005	28.6 ± 0.38	74.26 ± 3.27	0.64 ± 0.01
Scheme 5: HT (min): 5; ST (min): 10; SC (%, *w*/*v*): 2.5; LC (%, *w*/*v*): 5	DC: 0	88.7 ± 0.15	0.102 ± 0.013	35.3 ± 1.26	-	-
	DC: 0.5	100.4 ± 1.24	0.185 ± 0.022	30.6 ± 0.27	77.62 ± 1.36	0.67 ± 0.02
	DC: 1	114.9 ± 1.21	0.112 ± 0.005	32.3 ± 0.42	69.74 ± 1.03	0.81 ± 0.03
	DC: 1.5	126.2 ± 0.53	0.136 ± 0.014	29.3 ± 1.14	62.47 ± 1.04	0.92 ± 0.02
	DC: 2	231.3 ± 3.19	0.241 ± 0.043	29.7 ± 2.34	53.46 ± 1.93	1.15 ± 0.03
	DC: 2.5	252.7 ± 2.39	0.360 ± 0.037	26.1 ± 1.08	22.42 ± 1.37	1.15 ± 0.05

Abbreviation and unit of measurement: Homogenization time (HT) and sonication time (ST) measured in min, lipid concentration (LC), surfactant concentration (SC), and drug concentration (DC) measured in % *w*/*v*, polydispersity index (PI) is dimensionless, zeta potential (ZP) measured in mV, entrapment efficiency (EE) and drug loading (DL) measured in %.

**Table 2:** Effect of cryoprotectant on particle size, PI, ZP, EE and DL measured after reconstitution in double distilled water.

Process Optimized	Composition (% *w*/*v*)	Trehalose (% *w*/*v*)	Particle Size (nm)	PI	ZP (−mV)	EE (%)	DL (%)
HT: 5 min ST: 10 min	SC: 2.5 LC: 5 DC: 1	0	376.6 ± 4.58	0.427 ± 0.028	23.5 ± 0.32	Not detected	Not detected
		2.5	287.3 ± 2.98	0.317 ± 0.042	26.4 ± 0.41	57.46 ± 4.73	0.62 ± 0.04
		5	119.5 ± 2.17	0.124 ± 0.012	34.6 ± 0.32	71.43 ± 1.53	0.77 ± 0.03
		7.5	169.4 ± 3.08	0.236 ± 0.052	33.9 ± 0.18	56.29 ± 3.81	0.69 ± 0.04
		10	197.2 ± 3.81	0.257 ± 0.032	31.2 ± 0.46	55.37 ± 5.26	0.67 ± 0.05

## Data Availability

The data presented in this study are available on request from the corresponding author.

## References

[1] Afzali M., Ghaeli P., Khanavi M., Parsa P., Montazeri H., Ghahremani M.H., Ostad S.N. (2015). Non-addictive opium alkaloids selectively induce apoptosis in cancer cells compared to normal cells. J. Pharm. Sci..

[2] Awaad A.S., Maitland D.J., Moneir S.M. (2007). New alkaloids from *Casimiroa edulis* fruits and their pharmacological activity. Chem. Nat. Comp..

[3] Thandra K.C., Barsouk A., Saginala K., Aluru J.S., Barsouk A. (2021). Epidemiology of lung cancer. Contemp. Oncol..

[4] Abdul-Ghafar J., Oh S.S., Park S.M., Wairagu P., Lee S.N., Jeong Y., Eom M., Yong S., Jung S. (2013). Expression of adiponectin receptor 1 is indicative of favorable prognosis in non-small cell lung carcinoma. Tohoku. J. Exp. Med..

[5] Liu R.H. (2004). Potential synergy of phytochemicals in cancer prevention: Mechanism of action. J. Nutr..

[6] Obradovic A., Zizic J., Trisovic N., Bozic J., Uscumlic G., Bozic B. (2013). Evaluation of antioxidative effects of twelve 3-substituted-5,5-diphenylhydantoins on human colon cancer cell line HCT-116. Turk. J. Biol..

[7] Arap W.R., Pasqualini E., Ruoslahti (1998). Cancer treatment by targeted drug delivery to tumor vasculature in a mouse. Science.

[8] Satoskar R.R., Shah S.J., Shenoy S.G. (1986). Evaluation of anti-inflammatory property of curcumin (diferuloyl methane) in patients with postoperative inflammation. Int. J. Clin. Pharmacol. Ther. Toxicol..

[9] Negi P.S., Jayaprakasha G.K., Jagan Mohan Rao L., Sakariah K.K. (1999). Antibacterial activity of turmeric oil: A byproduct from curcumin manufacture. J. Agric. Food Chem..

[10] Lee Y.K., Park S.Y., Kim Y.M., Park O.J. (2009). Regulatory effect of the AMPK-COX-2 signaling pathway in curcumin-induced apoptosis in HT-29 colon cancer cells. Ann. N. Y. Acad. Sci..

[11] Kunnumakkara A.B., Anand P., Aggarwal B.B. (2008). Curcumin inhibits proliferation, invasion, angiogenesis and metastasis of different cancers through interaction with multiple cell signaling proteins. Cancer Lett..

[12] Li Y., Zhang J., Ma D., Zhang L., Si M., Yin H., Li J. (2012). Curcumin inhibits proliferation and invasion of osteosarcoma cells through inactivation of Notch-1 signaling. FEBS J..

[13] Chen H.W., Lee J.Y., Huang J.Y., Wang C.C., Chen W.J., Su S.F., Huang C.W., Ho C.C., Chen J.J.W., Tsai M. (2008). Curcumin inhibits lung cancer cell invasion and metastasis through the tumor suppressor HLJ1. Cancer Res..

[14] Zielinska A., Alves H., Marques V., Durazzo A., Lucarini M., Alves T.F., Morsink M., Willemen N., Eder P., Chaud M.V. (2020). Properties, extraction methods, and delivery systems for curcumin as a natural source of beneficial health effects. Medicina.

[15] Aggarwal B.B., Kumar A., Bharti A.C. (2003). Anticancer potential of curcumin: Preclinical and clinical studies. Anticancer. Res..

[16] Liu W., Zhai Y., Heng X., Che F.Y., Chen W., Sun D., Zhai G. (2016). Oral bioavailability of curcumin: Problems and advancements. J. Drug Target..

[17] Flora G., Gupta D., Tiwari A. (2013). Nanocurcumin: A promising therapeutic advancement over native curcumin. Crit. Rev. Ther. Drug Carr. Sys..

[18] Karthikeyan A., Senthil N., Min T. (2020). Nanocurcumin: A promising candidate for therapeutic applications. Front. Pharmacol..

[19] Gera M., Sharma N., Ghosh M., Huynh D.L., Lee S.J., Min T., Kwon T., Jeong D.K. (2017). Nanoformulations of curcumin: An emerging paradigm for improved remedial application. Oncotarget.

[20] Yadav P., Bandyopadhyay A., Chakraborty A., Sarkar K. (2018). Enhancement of anticancer activity and drug delivery of chitosan-curcumin nanoparticle via molecular docking and simulation analysis. Carbohydr. Polym..

[21] Sun J., Bi C., Chan H.M., Sun S., Zhang Q., Zheng Y. (2013). Curcumin-loaded solid lipid nanoparticles have prolonged in vitro antitumour activity, cellular uptake and improved in vivo bioavailability. Colloids Surf. B Biointerfaces.

[22] Shang L., Zhou X., Zhang J., Shi Y., Zhong L. (2021). Metal nanoparticles for photodynamic therapy: A potential treatment for breast cancer. Molecules.

[23] Basniwal R.K., Khosla R., Jain N. (2014). Improving the anticancer activity of curcumin using nanocurcumin dispersion in water. Nutr. Cancer.

[24] Chaurasia S., Chaubey P., Patel R.R., Kumar N., Mishra B. (2016). Curcumin-polymeric nanoparticles against colon-26 tumor-bearing mice: Cytotoxicity, pharmacokinetic and anticancer efficacy studies. Drug Dev. Ind. Pharm..

[25] Sun M., Su X., Ding B., He X., Liu X., Yu A., Lou H., Zhai G. (2012). Advances in nanotechnology-based delivery systems for curcumin. Nanomedicine.

[26] Pooja D., Tunki L., Kulhari H., Reddy B.B., Sistla R. (2015). Characterization, biorecognitive activity and stability of WGA grafted lipid nanostructures for the controlled delivery of rifampicin. Chem. Phys. Lipids.

[27] Chauhan H., Mohapatra S., Munt D.J., Chandratre S., Dash A. (2016). Physical-chemical characterization and formulation considerations for solid lipid nanoparticles. AAPS PharmSciTech.

[28] Joshi M., Patravale V. (2006). Formulation and evaluation of nanostructured lipid carrier (NLC)-based gel of valdecoxib. Drug Dev. Ind. Pharm..

[29] Chen C.C., Tsai T.H., Huang Z.R. (2010). Effects of lipophilic emulsifiers on the oral administration of lovastatin from nanostructured lipid carriers: Physicochemical characterization and pharmacokinetics. Eur. J. Pharm. Biopharm..

[30] Arya S.S., Rookes J.E., Cahill D.M., Lenka S.K. (2022). Reduced genotoxicity of gold nanoparticles with protein corona in *Allium cepa*. Front. Bioeng. Biotechnol..

[31] Florence A.T., Whitehill D. (1985). Stability and stabilization of water-in-oil-in-water multiple emulsions. Macro. Microemuls..

[32] Matsumoto S., Kita Y., Yonezawa D. (1976). An attempt at preparing water-in-oil-in-water multiple-phase emulsions. J. Colloid Inter. Sci..

[33] Das S., Chaudhury A. (2011). Recent advances in lipid nanoparticle formulations with solid matrix for oral drug delivery. AAPS PharmSciTech.

[34] Liu J., Hu W., Chen H., Ni Q., Xu H., Yang X. (2007). Isotretinoin-loaded solid lipid nanoparticles with skin targeting for topical delivery. Int. J. Pharm..

[35] Mcclements D.J. (2012). Crystals and crystallization in oil-in-water emulsions: Implications for emulsion-based delivery systems. Adv. Colloid Inter. Sci..

[36] Helgason T., Awad T.S., Kristbergsson K., McClements D.J., Weisse J. (2009). Effect of surfactant surface coverage on formation of solid lipid nanoparticles (SLN). J. Colloid Inter. Sci..

[37] Mehnert W., Mader K. (2001). Solid lipid nanoparticles: Production, characterization and applications. Adv. Drug Deliv. Rev..

[38] Shahgaldian P., Gualbert J., Aissa K., Coleman A.W. (2003). Study of the freeze-drying conditions of calixarene based solid lipid nanoparticles. Eur. J. Pharm. Biopharm..

[39] Kim B.D., Na K., Choi H. (2005). Preparation and characterization of solid lipid nanoparticles (SLN) made of cacao butter and curdlan. Eur. J. Pharm. Sci..

[40] Arya S.S., Rookes J.E., Cahill D.M., Lenka S.K. (2022). Chitosan nanoparticles and their combination with methyl jasmonate for the elicitation of phenolics and flavonoids in plant cell suspension cultures. Int. J. Biol. Macromol..

[41] Al-Jailawi M.H., Nasir H.M., Aziz G.M. (2015). Cytotoxic effect of bio-surfactants produced by novel thermophillic Geobacillus thermoleovorans (JQ 912239). Int. J. Adv. Res..

[42] Neves A.R., Lucio M., Lima J.L., Reis S. (2012). Resveratrol in medicinal chemistry: A critical review of its pharmacokinetics, drug-delivery, and membrane interactions. Curr. Med. Chem..

[43] Westesen K., Bunjes H. (1995). Do nanoparticles prepared from lipids solid at room temperature always possess a solid lipid matrix?. Int. J. Pharm..

